# Best practices for sharing information through data platforms: establishing the principles

**DOI:** 10.2471/BLT.16.172882

**Published:** 2016-04-01

**Authors:** Vasee S Moorthy, Cathy Roth, Piero Olliaro, Christopher Dye, Marie Paule Kieny

**Affiliations:** a Department of Immunization, Vaccines & Biologicals, World Health Organization, Geneva, Switzerland.; b Health Systems and Innovation Cluster, World Health Organization, avenue Appia 20, 1211 Geneva 27, Switzerland.; c Special Programme for Research and Training in Tropical Diseases (TDR), World Health Organization, Geneva, Switzerland.; d Department of Strategy, Policy & Information, World Health Organization, Geneva, Switzerland.

The public health benefits of data-sharing have been widely recognized over the past decade. A recent statement signed by over 30 research funders, nongovernmental organizations and publishers highlighted the importance of rapidly sharing information in public health emergencies.^1^ Several platforms for pre-publication data and manuscripts have been established. This journal has initiated Zika Open, a protocol for immediate posting of research manuscripts submitted in the context of the related public health emergency of international concern.

The demands of managing public health emergencies due to infectious disease outbreaks have prompted early sharing of data accruing from outbreak investigation and surveillance and public health-oriented research. Under the auspices of its blueprint for increasing research and development preparedness,^2^ and responding to the need for rapid and well-informed decision-making in emergencies, the World Health Organization (WHO) has developed a core set of principles for the sharing of data and results during public health emergencies. These principles were developed through an international multisectoral consultation held in Geneva in September, 2015.^3^ The increased provision of surveillance and research data means that central data repositories, inclusive of data curation services,^4^ are needed to provide the infrastructure for data sharing. 

In this editorial, we present a global health-oriented approach to the operation of data platforms, through three operating principles designed to reflect the rights and interests of all stakeholders while protecting the overarching public health objectives of the International Health Regulations (2005).

First, all data platforms should have an explicit ethical and legal framework governing data collection and use. The risks of withholding critical data need to be weighed against risks of sharing. Legitimate concerns regarding risks around personal data and the requirements of data protection legislation need to be addressed through reliable anonymization and encryption methods. Legal and ethical considerations also apply to publications and other products developed from the data. 

Second, results generated from additional analyses should be made public within a reasonable timeframe. The time required may vary depending on the situation, the complexity of the data, and the need for sufficient quality assurance. One perceived impediment to the early sharing of data is that the data providers may earn no credit for their work if analyses are first published by others. WHO considers that health research data are a global public good, but that data providers are also entitled to due credit for their work. Finding an effective way to assign credit and thus protect the interests of data providers – including government agencies, individual scientists, academic institutions and consortia – may determine the success of any data platform. Such recognition may range from acknowledgement to co-authorship or sharing of intellectual property, and should be determined in a fair and systematic fashion. Arrangements that concern data originating from low- and middle-income countries should include plans to build future local capacity for data management and analysis.

Third, platform operators should develop and publish terms for data-use, describing how each of the above principles will be applied to the data they receive, host and distribute. These terms should cover management of potential new intellectual property, and describe the process by which access to data is granted. The data platform operators are not the owners of the data, but fulfil important roles of stewardship and service provision.

Clinical trials present specific considerations because trial participants accept the unquantifiable risks of research in exchange for advances in knowledge. Ethical principles oblige rapid and full reporting of the data from clinical trials to prevent exposing additional participants to unnecessary risk. This reporting imperative is even more important in the context of a public health emergency. A three-step process for maximizing the utility of information from clinical trials could include universal prospective registration,^5^ timely public disclosure of results, and timely data sharing.

Data platforms can facilitate information sharing, and prior agreement on the principles set out here will speed the flow of information when it is most urgently needed, notably in public health emergencies. When put into practice, these principles will protect individuals' rights while maximising the substantial benefits for public health. WHO encourages all health data providers to adopt these principles and is working with Member States to implement them for data supplied in emergencies.

## References

[1] Global scientific community commits to sharing data on Zika. London: Welcome Trust; 2016. Available from: http://www.wellcome.ac.uk/News/Media-office/Press-releases/2016/WTP060169.htm [cited 2016 Mar 4].

[2] Modjarrad K, Moorthy VS, Millett P, Gsell P-S, Roth C, Kieny M-P. Developing Global Norms for Sharing Data and Results during Public Health Emergencies. PLoS Med. 2016 1;13(1):e1001935. 10.1371/journal.pmed.1001935 26731342 PMC4701443

[3] Developing global norms for sharing data and results during public health emergencies. Geneva: World Health Organization; 2015. Available from: www.who.int/medicines/ebola-treatment/data-sharing_phe/en/ [cited 2016 Mar 4]. 10.1371/journal.pmed.1001935 PMC4701443 26731342

[4] Powell-Smith A, Goldacre B. Explore England's prescribing data [Internet]. Oxford: EBM DATA LAB; 2015. Available from: www.openprescribing.net[cited 2016 Mar 4].

[5] Moorthy VS, Karam G, Vannice KS, Kieny M-P. Rationale for WHO’s new position calling for prompt reporting and public disclosure of interventional clinical trial results. PLoS Med. 2015 4;12(4):e1001819. 10.1371/journal.pmed.1001819 25874642 PMC4396122

